# CD63 negatively regulates hepatocellular carcinoma development through suppression of inflammatory cytokine‐induced STAT3 activation

**DOI:** 10.1111/jcmm.16167

**Published:** 2020-12-04

**Authors:** Shijun Yu, Jingde Chen, Ming Quan, Li Li, Yandong Li, Yong Gao

**Affiliations:** ^1^ Department of Oncology Shanghai East Hospital Tongji University School of Medicine Shanghai China

**Keywords:** CD63, hepatocellular carcinoma, migration, proliferation, STAT3

## Abstract

Tetraspanin CD63 has been widely implicated in tumour progression of human malignancies. However, its role in the tumorigenesis and metastasis of hepatocellular carcinoma (HCC) remains unclear yet. In the present study, we aimed to investigate the specific function and underlying mechanisms of CD63 in HCC progression. CD63 expression in HCC tissues was detected using immunohistochemistry and quantitative real‐time PCR analyses; effects of CD63 on HCC cell proliferation and migration were investigated by CCK‐8 assay, colony formation assay, transwell assay and a xenograft model of nude mice. RNA‐sequencing, bioinformatics analysis, dual‐luciferase reporter assay and Western blot analysis were performed to explore the underlying molecular mechanisms. Results of our experiments showed that CD63 expression was frequently reduced in HCC tissues compared with adjacent normal tissues, and decreased CD63 expression was significantly associated with larger tumour size, distant site metastasis and higher tumour stages of HCC. Overexpression of CD63 inhibited HCC cell proliferation and migration, whereas knockdown of CD63 promoted these phenotypes. IL‐6, IL‐27 and STAT3 activity was regulated by CD63, and blockade of STAT3 activation impaired the promotive effects of CD63 knockdown on HCC cell growth and migration. Our findings identified a novel CD63‐IL‐6/IL‐27‐STAT3 axis in the development of HCC and provided a potential target for the diagnosis and treatment of this disease.

## 1 INTRODUCTION

Hepatocellular carcinoma (HCC) is one of the most frequently diagnosed human malignancies worldwide, with approximately 841,080 new cases in 2018.^1^ Accumulated genetic mutations and epigenetic alterations induced by risk factors such as reactive oxygen species, inflammatory factors and fibrosis are related to the incidence and progression of HCC.^2^, ^3^ Although great developments in diagnosis and therapeutic strategies have occurred in recent years, such as hepatic resection, liver transplantation, transcatheter arterial chemoembolization (TACE) and multikinase inhibitor sorafenib, the long‐term survival rates of HCC remain dismal due to compensatory revascularization and drug resistance.^4^, ^5^ Therefore, it is necessary to explore the underlying mechanism of HCC pathogenesis to uncover new therapeutic strategies against HCC.

Tetraspanins are a group of cell surface‐associated membrane proteins expressed in a wide variety of cell types, and they are characterized by four transmembrane domains.^6^, ^7^ It is well established that tetraspanin proteins mediate cellular signal transduction and play important roles in the regulation of many physiological properties including cell motility, adhesion, proliferation, invasion and differentiation,^8^ and some tetraspanin molecules have been associated with progression of various human malignancies, such as melanoma, non‐small cell lung cancer (NSCLC), breast cancer and pancreatic cancer.^9^, ^10^ For example, tetraspanin CD82 is frequently down‐regulated in advanced stages of cancer, and overexpression of CD82 inhibits tumour migration and invasion via regulation of several signal pathways such as hepatocyte growth factor receptor (HGFR) pathway.^11^, ^12^ Tetraspanin CD9 has been reported to inhibit the proliferation and tumorigenicity of colon carcinoma cells,^13^ and ectopic expression of CD9 in NSCLC suppresses cell motility and promotes apoptotic cell death by regulating AKT phosphorylation and MMP2 secretion.^14^

The cluster of differentiation 63 (CD63), also known as melanoma‐associated antigen ME491 or MLA1, is the first identified member of the tetraspanin family.^15^ CD63 is ubiquitously expressed and mainly localized within the endosomal system and at the cell surface.^16^ Previous studies have implicated a role of CD63 in cellular transport of other proteins.^16^, ^17^, ^18^ For instance, it has been found in the complex with MHC II, which suggested that CD63 might chaperone MHCII molecules through the endosomal system and plays a role in antigen presentation via MHC II.^19^ More importantly, emerging evidences have implicated a correlation between abnormal CD63 expression and tumour progression. Although CD63 was first identified to be expressed in early‐stage melanoma cells, it is reduced while tumour cells become more invasive, implying a negative role of CD63 in tumour invasiveness.^20^ In other tumours including lung cancer and breast cancer, low CD63 expression is correlated with poor prognosis of patients.^21^ These findings suggested that CD63 is related to tumour cell motility and metastasis. However, its expression and functions in HCC remain to be elucidated.

In the present study, we resorted to investigate the role of CD63 in the development of HCC and explore the underlying molecular mechanisms using molecular and cell biology experiments in vitro and in vivo.

## 2 MATERIAL AND METHODS

### 2.1 Patients and HCC tissue samples

Primary human HCC tissues and paired adjacent normal tissues were collected from 35 patients at Shanghai East Hospital, Tongji University School of Medicine, China. All the specimens were snap‐frozen in liquid nitrogen immediately after resection for future use. Informed consent was obtained from each patient, and the use of the samples for the present study has been approved by the ethics committee of Shanghai East Hospital, Tongji University School of Medicine, China.

### 2.2 Tissue microarray (TMA) and immunohistochemistry

For immunohistochemistry analysis, a TMA containing 75 paired human HCC tissues and adjacent normal tissues was purchased from Shanghai Outrdo Biotech (#LivH150CS03). The clinicopatholofical information of the patients can be checked at the website: http://www.superchip.com.cn/biology/tissue.html. Human antibody against CD63 (#sc‐15363, Santa Cruz Biotechnology, USA) was used to perform immunohistochemistry analysis according to the standard protocol of manufacturer (Outdo Biotech, Shanghai, China), respectively. The staining results were analysed by two pathologists blinded to the clinical information as described previously.^22^

### 2.3 Cell culture and reagents

Human HCC cell lines Huh7, L02, MHCC‐LM3 and PLC/PRF/5 were purchased from the Shanghai Cell Bank of the Chinese Academy Sciences, Shanghai, China. Cells were maintained in Dulbecco's Modified Eagle Medium (DMEM, Corning, Inc) supplemented with 10% foetal bovine serum (FBS, Corning, Inc) and 1% penicillin/streptomycin (M&C Gene Technology Ltd.) and incubated at 37 ˚C with 5% CO_2_. STAT3‐specific inhibitor BP‐1‐102 was purchased from Selleckchem, Houston, TX, USA.

### 2.4 RNA isolation and quantitative real‐time polymerase chain reaction (qPCR) analysis

Total RNAs were extracted with TRIzol reagents from Sigma‐Aldrich, Merck KGaA, Germany according to the protocol of manufacturer and reverse transcribed into cDNA using the Primescript™ RT Reagent kit with gDNA Eraser (Takara) and stored at −20°C before use. Standard qPCR analysis was performed with SYBR‐Green reagents (Takara) on an ABI QuantStudio™ 6 Flex system according to the specifications of manufacturers. 2^ΔΔCt^ method was used to calculate relative mRNA levels of target genes, and β‐actin was used endogenous control. The following primer sequences were used: CD63 forward: 5′‐TGGAAGGAGGAATGAAATGTG‐3′, reverse: 5′‐TCTTCTCCATACCAGCTTCCTT‐3′; IL‐6 forward: 5′‐ACCCCTGACCCAACCACAAAT‐3′, reverse: 5′‐AGCTGCGCAGAATGAGATGAGTT‐3′; IL‐27 forward: 5′‐ACCGCTTTGCGGAATCTCA‐3′, reverse: 5′‐AGGTCAGGGAAACATCAGGGA‐3′; β‐actin forward: 5′‐CCTGGCACCCAGCACAATG‐3′, reverse: 5′‐GGGCCGGACTCGTCATACT‐3′.

### 2.5 Cell transfection

For CD63 overexpression, pEnter‐CD63 plasmid (GeneBank Accession Number: NM_001780) was purchased from Vigene Biosciences, Shandong, China. Specific small inference RNA against CD63 (siCD63) and non‐specific control (siNC) were purchased from Shanghai Genepharma Company. The sequences were as follows: siCD63 sense 5′‐GUGGGAUUAAUUUCAACGA‐3′; siNC sense 5′‐UUCUCCGAACGUGUCACGUdTdT‐3′. Transfection of siRNAs or plasmids was carried out using Lipofectamine 3000 Reagents (Introgen) according to the protocol of manufacturer. To establish stably infected cell lines with CD63 overexpression or knockdown, RNA inference lentivirus (sequence of siCD63 was used) and CD63 overexpression lentivirus were purchased from GenePharma, Shanghai China. Cells were infected with the indicated lentiviruses with the assistance of polybrene (4 μg/mL), and stably infected cells were selected with puromycin (2.5 μg/mL).

### 2.6 Western blot analysis

Total proteins were isolated from HCC cells with RIPA Lysis Buffer (Beyotime, Jiangsu, China). After centrifugation at the speed of 14000 *g* for 10 minutes at 4°C, cell lysates were resolved by 5% SDS loading buffer and boiled for 5 minutes. Equal amounts of cell lysates were electrophoresed on 10% SDS‐PAGE and transferred to a polyvinylidene difluoride (PVDF) membrane (Bio‐Rad Laboratories). After blocking in 5% non‐fat dry milk supplemented with 0.1% Tween‐20 in PBS (PBST) at room temperature for 1 hours, the membrane was incubated with specific primary antibodies overnight at 4°C followed by incubation with corresponding secondary antibodies for 1 hour. The immunoreactive signal was visualized using the Odyssey Infrared imaging system (LI‐COR Biosciences). The antibodies used in the present study included: anti‐CD63 (1:200, #sc‐15363, Santa Cruz Biotechnology, USA), anti‐STAT3 (1:2000, #9139, Cell Signaling Technology), anti‐pSTAT3 (Y705, 1:500, #9145, Cell Signaling Technology) and anti‐β‐actin (1:1,000, #81178, Santa Cruz Biotechnology).

### 2.7 Dual‐luciferase reporter assay

Briefly, HCC cells were seeded into a 24‐well plate and incubated for 24 hours before transfection, then 250 ng of STAT3 reporter plasmid (Genomeditech) and 10 ng of SV40, a Renilla luciferase expression plasmid as an internal control were cotransfected into the prepared cells. 24 hours after transfection, cells were harvested and dual‐luciferase reporter assay was carried out with a Dual‐Glo Luciferase kit according to the manufacturer's specifications (Promega). A GloMax^®^ 96 Microplate Luminometer (Promega) was used to quantify both firefly and Renilla luciferase activities. All experiments were done in triplicate and repeated at least 3 times.

### 2.8 RNA‐sequencing and bioinformatics analysis

Briefly, Huh7 cells were seeded into a 10‐cm dish at a confluence of 80% and incubated for 24 hours, after which total RNA was extracted with TRIzol reagents (Sigma‐Aldrich, Merck KGaA) according to the protocol of manufacturer and immediately snap‐frozen in liquid nitrogen before RNA‐sequencing. The RNA‐sequencing was performed on a BGISEQ‐500 platform and mapped by BGI. The log_2_ ratio of fragments per kilobase of transcript per million (FPKM) between Huh7‐VEC and Huh7‐CD63 cells was calculated and genes with an absolute value of log_2_FPKM ≥ 1 or ≤−1 and an adjusted *P* value (*q* value) <.05 were considered to be differentially expressed genes. Volcano plot exhibiting differentially expressed genes were made using ggplot2 package in R language. Kyoto Encyclopedia of Genes and Genomes (KEGG) pathway analysis and Gene Set Enrichment Analysis (GSEA) were performed to analyse the pathways which the down‐regulated genes in Huh7‐CD63 cells were enriched in.

### 2.9 CCK‐8 cell proliferation assay

Cell Counting Kit‐8 reagent (CCK‐8, Dojindo Laboratories) was used to perform cell proliferation assays. Briefly, the indicated cells were seeded into 96‐well plates in triplicate (3000 cells per well) after transfection. At scheduled time points (0, 24, 48, 72 and 96 hours), 10 μL of CCK‐8 was added into each well and incubated for 1 hour at 37°C. An automated plate reader (SpectraMax M5, Molecular Devices, LLC) was used to read the absorbance at a wavelength of 450 nm. Each experiment was performed in triplicate and repeated 3 times independently.

### 2.10 Colony formation assay

For colony formation assays, stably infected HCC cells were cultured in a 6‐well plate at a density of 1000 cells per well and incubated at 37°C for 1‐2 weeks; then, the colonies were harvested. After fixation in 4% paraformaldehyde, the colonies were stained with crystal violet (Sangon Biotech) for 10 minutes. Images of the colonies were captured, and the number of cells was counted using Image J software.

### 2.11 Cell migration assay

Cell migration assays were performed with a 24‐well transwell chamber (pore size, 8 μm; Costar). Briefly, 30 000 HCC cells suspended in 400 μL DMEM without FBS were added into the upper chamber, while 800 μL of DMEM containing 10% FBS was added into the bottom chamber. After incubating at 37°C for 48 hours, the migrated cells were harvested and stained with crystal violet (Sangon Biotech) for 20 minutes. A phase‐contrast microscope (Leica DM6000B, Leica Microsystems) was used to photograph and count the number of migrated cells in five randomly selected fields at a magnification of ×100.

### 2.12 Animal experiments

To establish a xenograft model of HCC, 4‐ to 6‐week‐old male BALB/c nude mice were purchased from Sippr‐BK laboratory animal corporation, Shanghai, China. Equal amount of stably infected HCC cells (2 × 10^6^ cells) were injected subcutaneously into each flank of armpit area of nude mice, respectively (n = 5). After 4 weeks, the mice were euthanized, and each tumour was weighted after resection. The animal handling and experimental procedures were abided by the Ethics Committee of Shanghai East Hospital, Tongji University School of Medicine.

### 2.13 Enzyme‐linked immunosorbent assay (ELISA)

To evaluate the effects of CD63 on IL‐6 and IL‐27 protein levels, the indicated HCC cells were treated with LPS (1 μg/mL, Beyotime) for 6 hours to induce the production of inflammatory cytokines and the culture supernatants were collected. IL‐6 and IL‐27 protein levels were measured using ELISA kits (#KE00007 and #KE00089, Proteintech) according to the manufacturer's instructions.

### 2.14 Statistical analysis

Quantitative data were represented as the means ± SD, and GraphPad Prism software 7.0 was used to perform statistical analyses. The chi‐square test was used to explore the relationships between CD63 expression and clinicopathological parameters of HCC patients. Statistical significance was determined by one‐way analysis of variance (one‐way ANOVA), and Dunnett's multiple comparison test was used as a post hoc test or the Student *t* test. A *P* value less than 0.05 was considered as statistically significant. *P < .05, **P < .01.

## 3 RESULTS

### 3.1 CD63 expression is reduced in HCC tissues and associated with clinicopathological parameters of HCC patients

**FIGURE 1:**
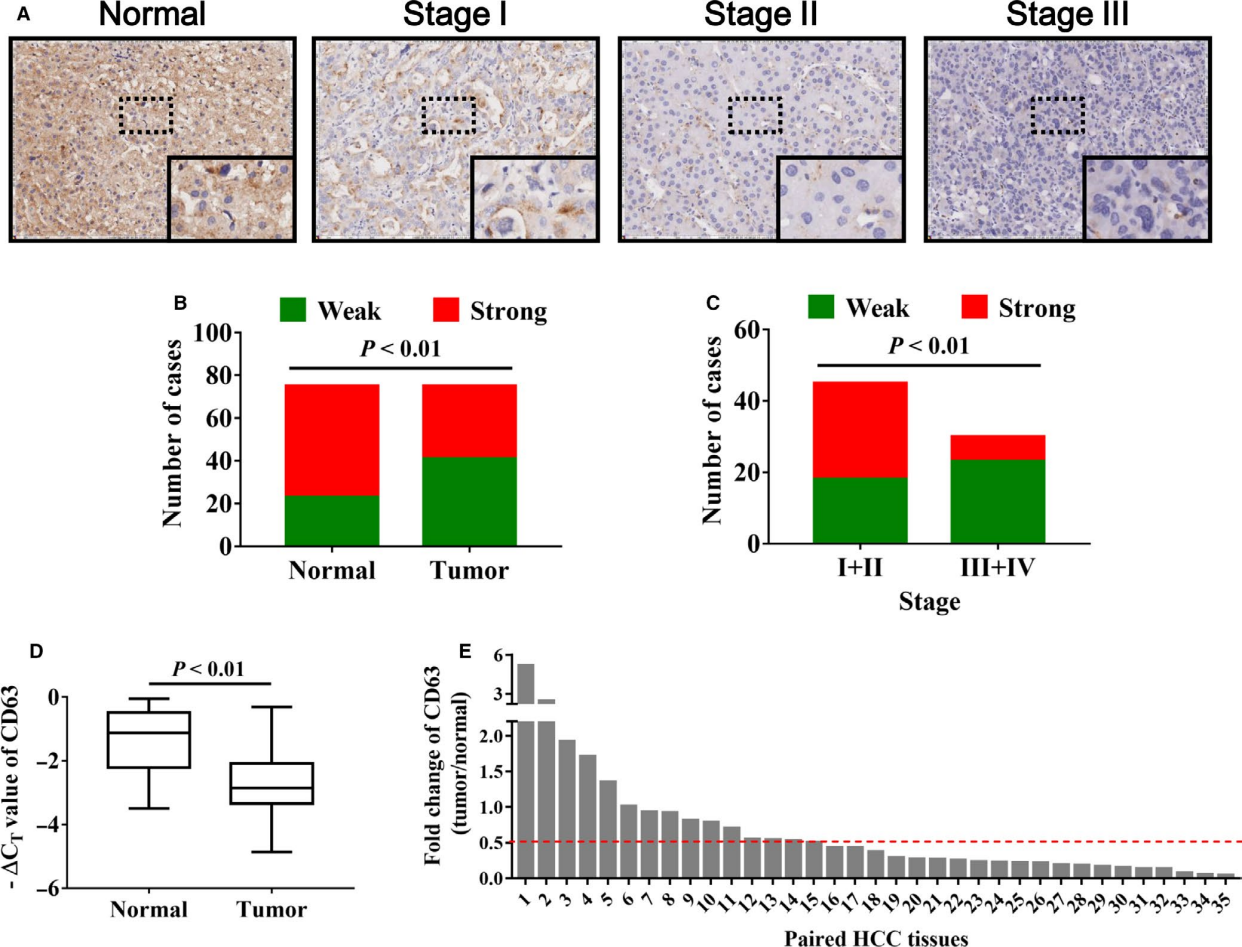
CD63 expression is significantly decreased in HCC tissues. A, immunohistochemistry analysis was performed to detect CD63 protein expression in HCC tissues, and representative images of CD63 in different stages of HCC were shown. Magnification: 40× (upper), 200× (bottom). B, the staining results of immunohistochemistry analysis were shown in the column diagram. C, negative correlation between CD63 expression and tumour stages of HCC was shown. D, the mRNA levels of CD63 in 35 pairs of HCC tissues and adjacent normal tissues were evaluated by quantitative real‐time PCR analysis, and ‐ΔCT value represented relative expression of CD63 mRNA. E, the box plot indicated the fold changes of CD63 mRNA expression (tumour/normal) in HCC tissues

The protein expression of CD63 in a tissue microarray (TMA) consisting of 75 HCC tumour tissues and paired adjacent normal tissues was analysed by immunohistochemistry. The results showed that CD63 was frequently down‐regulated in tumour tissues compared with adjacent normal tissues at protein level (Figure 1A,B). Next, we analysed the relationship between CD63 expression and clinicopathological parameters in the TMA. Lower CD63 expression was significantly associated with larger tumour size (*P* < .05), distant site metastasis (*P* < .01) and higher TNM stage (*P* < .01) (Table 1 and Figure 1C). Furthermore, the mRNA level of CD63 in additional 35 paired HCC tissues was determined by qPCR analysis. Consistently, CD63 mRNA expression in tumour tissues was remarkably lower than in adjacent normal tissues (Figure 1D), and it exhibited a <0.5‐fold decrease in 21/35 (60%) paired tissues (Figure 1E). Taken together, these results suggested that CD63 expression is reduced in HCC tissues and directly associated with the development of HCC.

**Table 1:** The correlation of CD63 expression with clinicopathological features of HCC

	Case number	Weak staining	Strong staining	*P* value
Age (y)				
>60	18	10	8	.931
≤60	57	31	26	
Gender				
Male	62	33	29	.584
Female	13	8	5	
Liver cirrhosis				
No	27	15	12	.908
Yes	48	26	22	
Tumour size				
<5 cm	36	14	22	.008*
≥5 cm	39	27	12	
Metastasis				
M0	71	37	34	.009*
M1	4	4	0	
AJCC stage				
I, II	45	18	27	.002*
III, IV	30	23	7	
Total	75	41	34	

* *P* < .05 was considered as a significant association among the variables.

### 3.2 CD63 inhibits HCC cell proliferation in vitro and tumorigenicity in animal models

**FIGURE 2:**
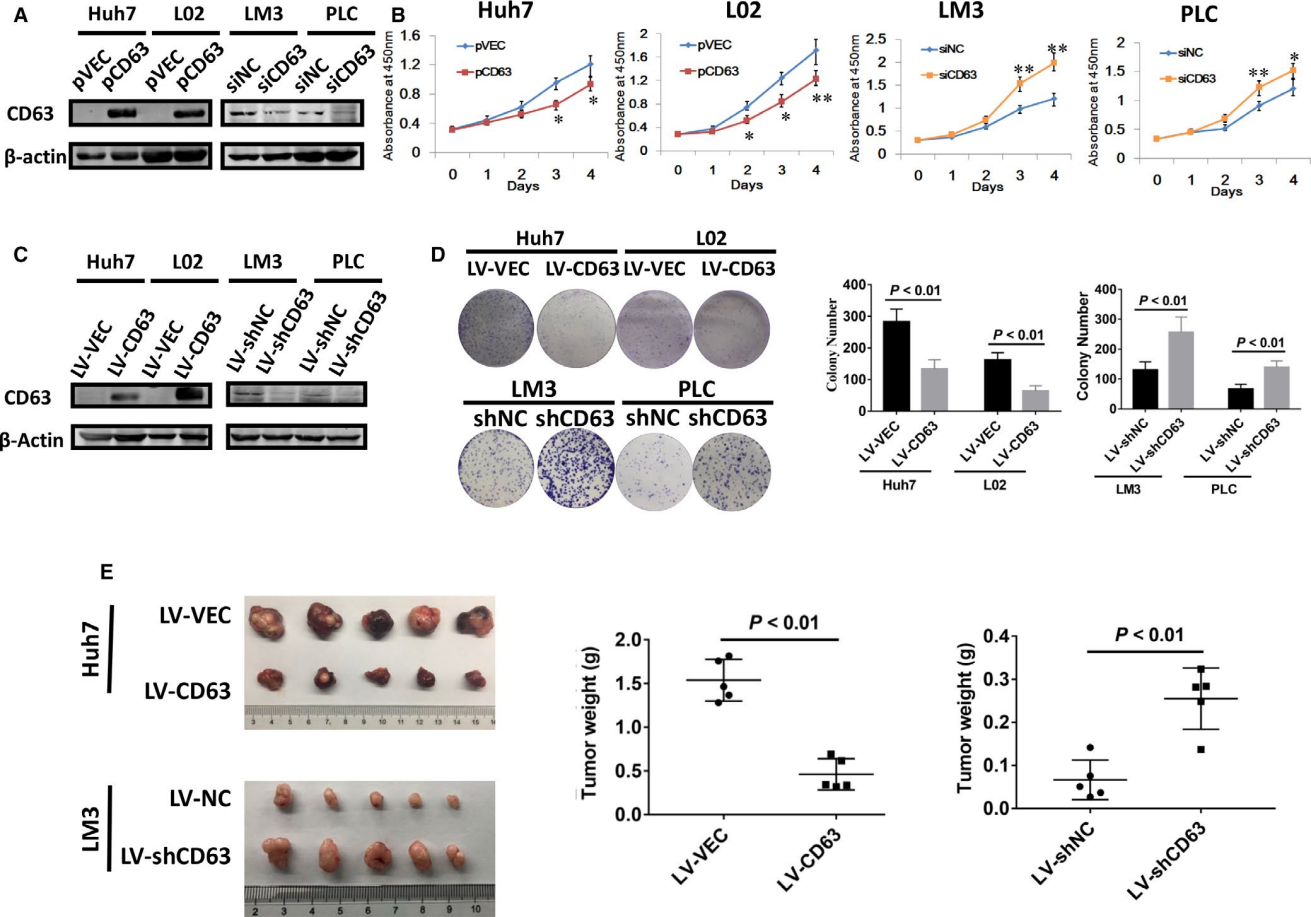
CD63 acts as a tumour suppressor gene in HCC cell proliferation in vitro and in vivo. A, 48 h after cell transfection, the efficacies of CD63‐expressing plasmids (pCD63) and siRNAs targeting CD63 (siCD63) were confirmed by Western blot analysis, respectively. Empty vector (pVEC) and non‐specific control siRNA (siNC) were used as control group. B, proliferative abilities of HCC cells were evaluated using CCK‐8 assays. C, CD63 expression in stably infected HCC cells were examined by Western blot analysis. D, colony formation assays were performed using stably infected HCC cells. E, Huh7 and MHCC‐LM3 cells infected with LV‐CD63/LV‐shCD63 were subcutaneously inoculated into nude mice, respectively. Cells infected with LV‐VEC/LV‐shNC were used as control group. (n = 5 per group). Tumours were resected and weighted after 4 wk. *P < .05, **P < .01

To explore the impacts of CD63 on cell proliferation of HCC, we intervened its expression by transfecting CD63‐expressing plasmids (pCD63) or siRNAs targeting CD63 (siCD63) into HCC cell lines, respectively. Western blot results confirmed the efficiencies of pCD63 and siCD63 (Figure 2A). As the same time, CCK‐8 assays were employed to detect cell proliferation. As shown in Figure 2B, HCC cells transfected with pCD63 exhibited a significantly lower growth rate comparing with the control group, whereas knockdown of CD63 by siCD63 resulted in opposite results. Furthermore, CD63 expression was stably overexpressed or silenced by transducing the HCC cells with lentiviruses (LV‐CD63 or LV‐shCD63, Figure 2C), and colony formation assays were conducted. In line with the results of CCK‐8 assays, colonies formed from cells with increased CD63 expression were significantly less and smaller than those from the control cells, while blockade of CD63 attenuated colony formation capacity of HCC cells (Figure 2D). In addition, a xenograft mice model was established to validate these findings in vivo. As expected, CD63 overexpression significantly inhibited tumour growth of Huh7 cells, while knockdown of CD63 promoted tumorigenicity of MHCC‐LM3 cells (Figure 2E). Therefore, these results demonstrated that CD63 plays a critical role in suppressing HCC cell proliferation in vitro and in vivo.

### 3.3 CD63 negatively regulates HCC cell migration in vitro

**FIGURE 3:**
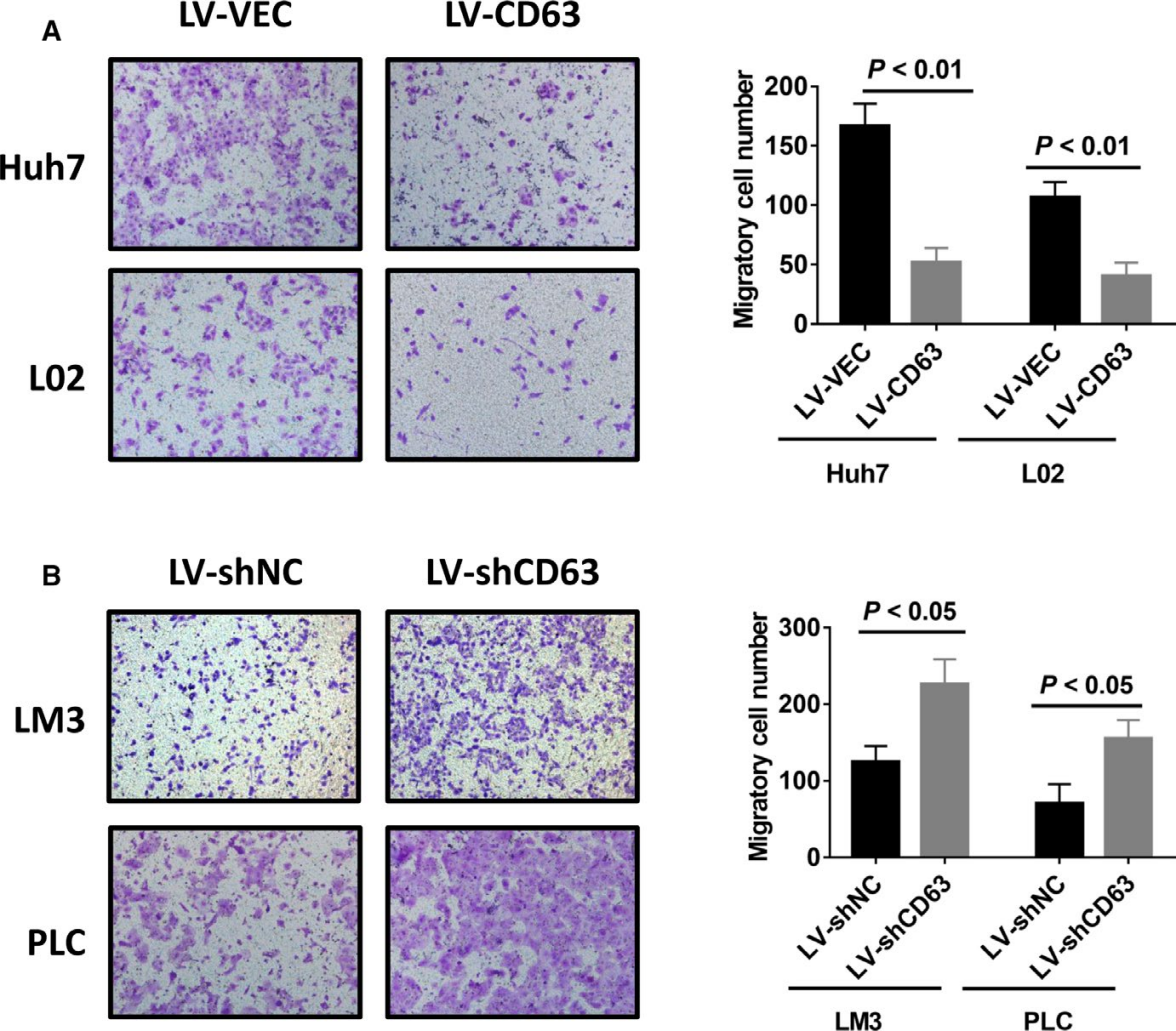
Inhibition of HCC cell migration by CD63 in vitro. A and B, transwell migration assays were performed using stably infected HCC cells. Representative images of migratory cells (left) and graphs (right) showing the suppression of CD63‐overexpression (A) and enhancement of CD63‐knockdown (B) in migration ability of HCC cells were presented

In previous studies, CD63 has been associated with tumour metastasis in other tumours including melanoma and breast cancer.^20^, ^23^ Therefore, we next examined whether CD63 could influence HCC cell migration ability in vitro. After intervening CD63 expression in the indicated HCC cell lines, transwell migration assays were performed. As presented in Figure 3A,B, overexpression of CD63 significantly reduced the migratory cell number of Huh7 and L02 cells. In contrast, CD63 silencing strongly promoted cell migration ability of MHCC‐LM3 and PLC/PRF/5 cells. Thus, our results suggested a negative role of CD63 in HCC cell migration.

### 3.4 CD63 inhibits inflammation‐related oncogenic signalling pathways in HCC cells

**FIGURE 4:**
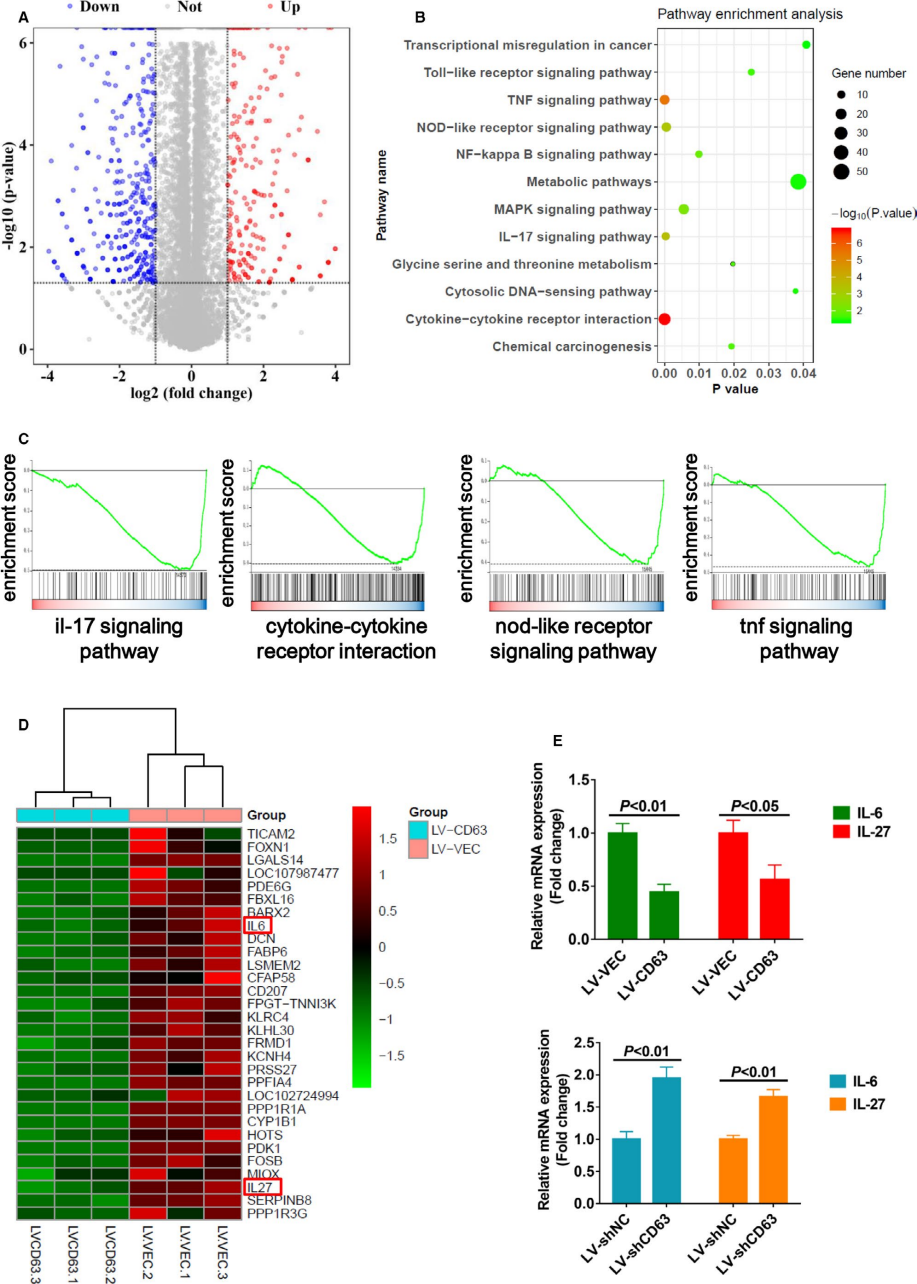
CD63 inhibits activation of inflammation‐related oncogenic pathways in HCC cells. A, volcano plot showed differentially expressed genes between Huh7‐CD63 cells and control Huh7 cells. Red dots indicated up‐regulated genes in Huh7‐CD63 cells, and blue dots indicated down‐regulated genes in Huh7‐CD63 cells. Fold change ≥2 or ≤−2 and *q* value <0.05 were considered as effective candidates. B, 539 down‐regulated genes in Huh7‐CD63 cells were used to perform KEGG pathway analysis. Gene numbers enriched in each pathway were exhibited as different size of circles. C, results of GSEA analysis showed that IL‐17 signalling pathway, cytokine‐cytokine receptor interaction pathway, NOD‐like receptor signalling pathway and TNF signalling pathway were enriched in control Huh7 cells comparing with Huh7‐CD63 cells. D, heat map was used to show the top 30 down‐regulated genes in huh7‐CD63 cells. E, after transfection with pCD63 or siCD63, qPCR analysis was performed to detect the mRNA levels of cytokines IL‐6 and IL‐27 in HCC cells

To explore the molecular mechanisms through which CD63 inhibited HCC cell proliferation and migration, RNA‐sequencing was applied using Huh7 cells with ectopic CD63 expression, and global transcriptional expression profile differences were analysed. Volcano plot showed that 393 genes were up‐regulated and 539 genes were down‐regulated in cells with CD63 overexpression when compared with control cells (Figure 4A). Furthermore, the down‐regulated genes were used to perform KEGG pathway analysis. The results showed that the down‐regulated genes were highly enriched in inflammation‐related oncogenic signalling pathways, such as IL‐17 signalling pathway, NF‐κB signalling pathway and NOD‐like receptor signalling pathway (Figure 4B). Besides, the gene set enrichment analysis (GSEA) was used to verify these results. Compared to the control group, increased CD63 expression significantly inhibited the activities of those inflammation‐related oncogenic signalling pathways (Figure 4C). Therefore, these in‐silico findings suggested that the tumour‐suppressive effects of CD63 might be mediated by inflammation‐related oncogenic signalling pathways. In order to reveal the critical genes by which CD63 regulated these pathways, we analysed the list of top 30 down‐regulated genes. Intriguingly, interleukin‐6 (IL‐6) and interleukin‐27 (IL‐27) were dramatically down‐regulated after CD63 overexpression (Figure 4D), both of which are involved in the regulation of inflammation in the tumour microenvironment as well as proliferation, survival and invasiveness of tumour cells through downstream signalling pathways such as JAK/STAT3 pathway.^24^, ^25^, ^26^ More importantly, qPCR results indicated that forced expression of CD63 significantly inhibited IL‐6 and IL‐27 expression, whereas knockdown of CD63 dramatically increased the levels of IL‐6 and IL27 in HCC cells (Figure 4E). Likewise, a substantial increase of IL‐6 and IL‐27 protein levels was observed in culture supernatants of MHCC‐LM3 cells with silenced CD63 expression by ELISA (Figure S1). The results mentioned above suggested that CD63 may suppress HCC cell proliferation and migration possibly by inhibiting the expression of cytokines IL‐6 and IL27, which in turn inactivate inflammation‐related oncogenic signalling pathways.

### 3.5 CD63 has a negative impact on STAT3 activation in HCC

**FIGURE 5:**
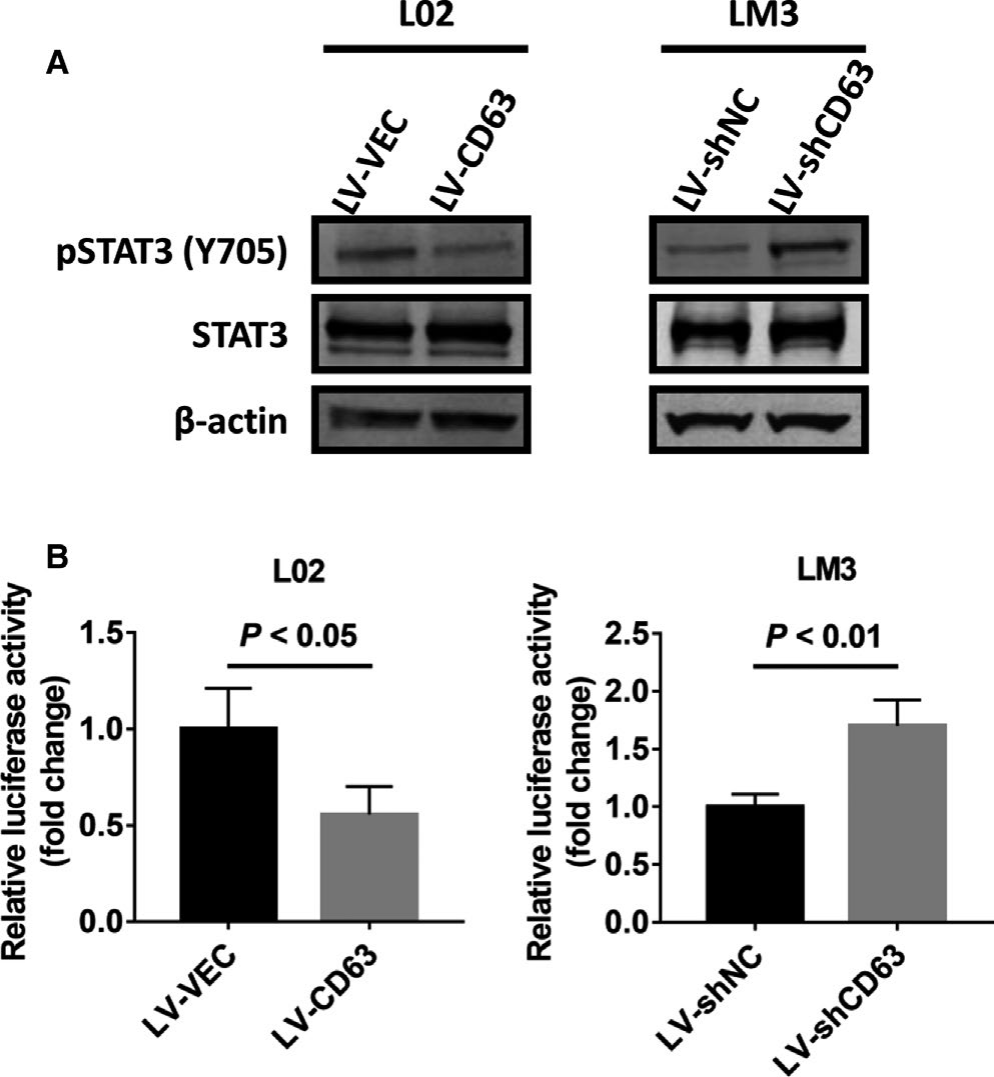
CD63 suppresses STAT3 activation in HCC cells. A, Western blot analysis were performed to detect the expression of p‐STAT3 (Y705) and total STAT3 in HCC cells stably infected with LV‐CD63 or LV‐shCD63. LV‐VEC and LV‐shNC were used as control groups, respectively. B, 250 ng of the STAT3 response element reporter plasmid was transfected into the indicated HCC cells, respectively. After incubation for 24 h, dual‐luciferase reporter assays were performed to determine the transcriptional activity of STAT3

Considering that both IL‐6 and IL‐27 contribute to tumour progression through JAK/STAT3 signalling pathway,^24^, ^25^ we next elucidated whether CD63 regulated the activation of signal transducer and activator of transcription‐3 (STAT3) by Western blot analysis. As shown in Figure 5A, overexpression of CD63 significantly decreased the level of phosphorylated STAT3 (Y705), while total protein levels of STAT3 showed no obvious difference. In contrast, silencing CD63 expression led to opposite results. To verify this regulation of STAT3 by CD63, a dual‐luciferase reporter assay was performed using STAT3 response element reporter plasmid. As expected, overexpression of CD63 inhibited the transcriptional activity of STAT3 in L02 cells, while knockdown of CD63 remarkably increased the transcriptional activity of STAT3 in MHCC‐LM3 cells (Figure 5B). These mechanistic studies demonstrated that CD63 could inactivate STAT3 in HCC cells.

### 3.6 STAT3 inhibition blocks CD63 knockdown‐induced cell growth and migration

**FIGURE 6:**
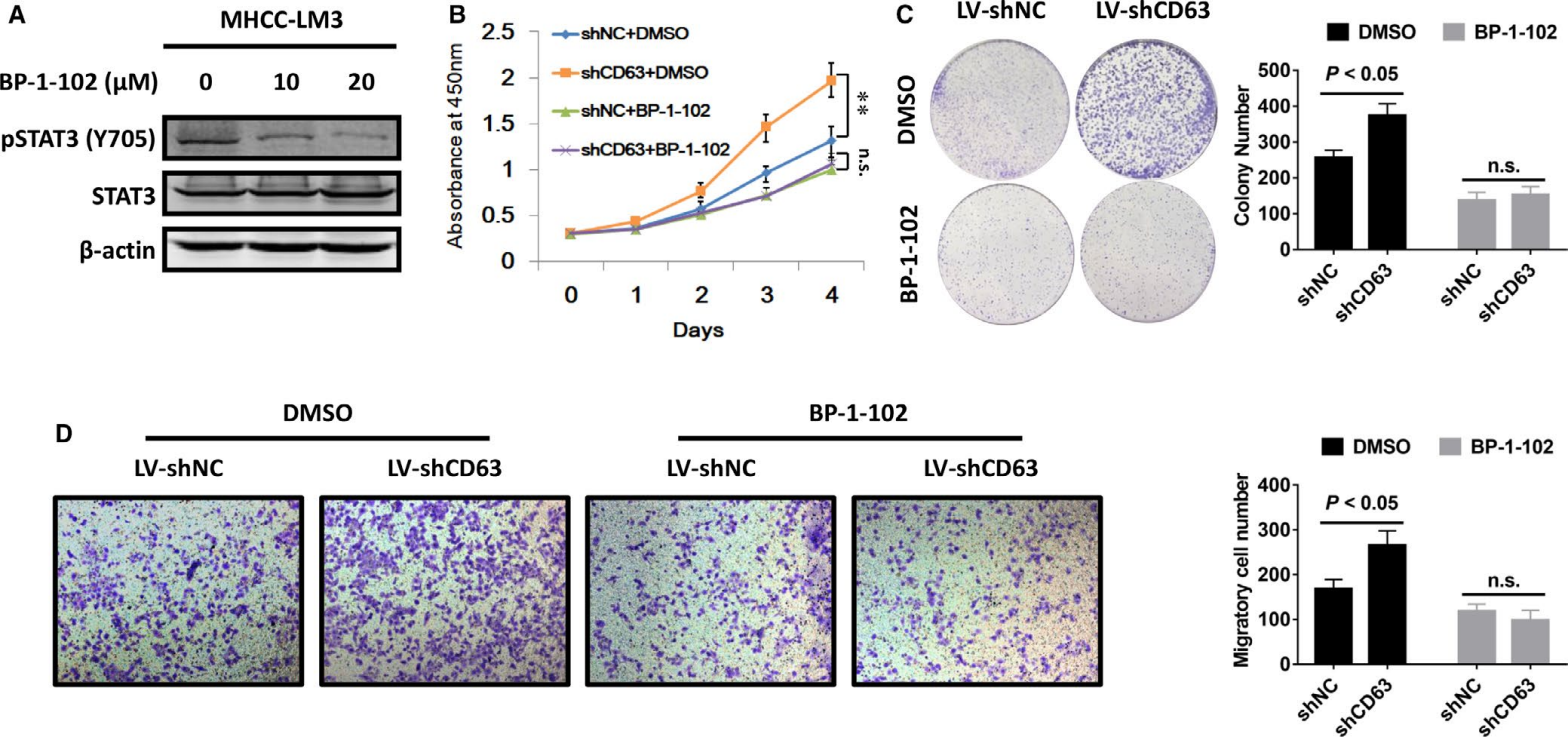
STAT3 signalling acts downstream of CD63 in HCC. A, MHCC‐LM3 cells were treated with BP‐1‐102 at the indicated concentrations for 12 h, and Western blot analysis was conducted to determine the changes of p‐STAT3 (Y705) and total STAT3 expression. B and C, the indicated HCC cells were cultured in medium supplemented with BP‐1‐102 (10 μmol/L) or DMSO, and CCK‐8 assays and colony formation assays were carried out to assess the influence of BP‐1‐102 on the effects of CD63 silencing in proliferation ability of MHCC‐LM3 cells. D, after treating the above HCC cells with BP‐1‐102 (10 μmol/L) or DMSO, transwell migration assays were performed to confirm whether CD63 inhibited HCC cell migration via inactivating STAT3

To assess the effects of the relationship between CD63 and STAT3 on cell growth and migration in HCC, a STAT3‐specific inhibitor BP‐1‐102 was used to block STAT3 activation^27^ (Figure 6A). Results of CCK‐8 and colony formation assays showed that treatment with BP‐1‐102 significantly abolished CD63 knockdown‐induced cell growth and colony formation of MHCC‐LM3 cells (Figure 6B,C). Similarly, the data from transwell migration assays also demonstrated that BP‐1‐102 treatment dramatically attenuated the migratory ability of CD63 silenced MHCC‐LM3 cells (Figure 6D). These findings collectively hinted us that STAT3 signalling is required for the negative effects of CD63 on cell proliferation and migration in HCC.

## 4 DISCUSSION

Although previous studies have improved early diagnosis and treatment of HCC, the underlying mechanisms of initiation and development are largely unknown, which are critical for the development of effective interventions against HCC. In the present study, we demonstrated for the first time that tetraspanin CD63 is a potential tumour suppressor in HCC. Our clinical investigation showed that CD63 expression was frequently down‐regulated in HCC tissues, and reduced CD63 expression was associated with larger tumour size, distant site metastasis and higher TNM stages.

CD63 is an important member of the tetraspanin superfamily involved in the regulation of a variety of cellular physiological activities such as membrane protein trafficking, cell fusion, adhesion, motility and cell differentiation, and abnormal CD63 expression has been associated with different human malignancies. Consistent with some previous reports,^20^, ^21^, ^28^ our findings revealed that CD63 inhibits HCC cell proliferation and migration in vitro, and results of animal experiments confirmed its negative role in tumour growth in vivo. Further RNA‐sequencing and bioinformatics analysis suggested that overexpression of CD63 in HCC cells significantly inhibited the activities of inflammation‐related oncogenic signalling pathways including IL‐17 signalling pathway, NF‐κB signalling pathway and NOD‐like receptor signalling pathway, which are known to stimulate inflammation and inhibit immunoreaction in tumour microenvironments, thus promoting initiation and progression of various tumours.^29^, ^30^, ^31^ Importantly, we identified that cytokines IL‐6 and IL‐27 are downstream targets of CD63, as evidenced by the results of RNA‐seq and qPCR analysis that overexpression of CD63 significantly inhibited IL‐6 and IL‐27 expression in HCC cells.

Elevated levels of IL‐6 and IL‐27 have been observed in chronic inflammatory conditions, such as rheumatoid arthritis and a lot of hematopoietic malignancies and solid tumours.^32^, ^33^ In the pathogenesis of cancer, elevated IL‐6 and IL‐27 could stimulate the activation of JAK1 and JAK2 enzymes, which subsequently mediate tyrosine phosphorylation of STAT3 at Tyr705. After translocation into nucleus, activated STAT3 regulates transcription of its target genes encoding regulators of cellular proliferation and survival, such as cyclin D1 and survivin.^24^, ^34^ Thus, targeting hyperactivated IL‐6/IL‐27‐JAK‐STAT3 pathway is beneficial in the treatment of certain cancers. Our results demonstrated that CD63 inhibited STAT3 activation possibly through inhibiting IL‐6 and IL‐27 expression. Further cell biology experiments confirmed that CD63 may exert its function via inhibiting STAT3 activity. Interestingly, another study of osteosarcoma also reported that knockdown of CD63 resulted in decreased pY705‐STAT3 expression,^35^ which supports our findings in HCC. Therefore, these data establish a CD63‐IL‐6/IL‐27‐STAT3 axis involved in the regulation of HCC cell proliferation and migration. However, it is still unknown how CD63 regulates IL‐6 and IL‐27 expression in HCC cells.

It is well established that CD63 is an important biomarker of exosomes, which are microvesicles secreted by most types of cells and participate in intercellular communication via transmitting its cargo including proteins and nucleic acids into receptor cells.^36^, ^37^ Intriguingly, Cheng et al reported that a specific type of exosomes (p120ctn) inhibited HCC cell progression via STAT3 pathway, hinting us that CD63 possibly regulates inflammation‐related oncogenic pathways by influencing biogenesis and secretion of exosomes.^38^ However, more studies need to be done in order to verify this hypothesis.

In conclusion, these clinical and mechanistic findings identified a novel CD63‐IL‐6/IL‐27‐STAT3 axis in HCC tumorigenesis and development, and targeting this axis is a potential therapeutic strategy against HCC.

## CONFLICT OF INTEREST

The authors declare there is no conflict of interest.

## AUTHOR CONTRIBUTION

**Shijun Yu:** Data curation (equal); Investigation (equal); Methodology (equal); Writing‐original draft (lead). **Jingde Chen:** Data curation (equal); Formal analysis (equal); Investigation (equal); Methodology (equal). **Ming Quan:** Conceptualization (equal); Data curation (equal); Formal analysis (equal). **Li Li:** Data curation (equal); Formal analysis (equal); Funding acquisition (equal). **Yandong Li:** Investigation (equal); Project administration (equal); Supervision (equal); Validation (equal). **Yong Gao:** Investigation (equal); Project administration (equal); Supervision (equal); Writing‐review & editing (equal).

## ETHICAL APPROVAL

This study has been approved by the ethics committee of Shanghai East Hospital, Tongji University School of Medicine, China.

## Supporting information

Fig S1

## Data Availability

The data used to support the findings of this study are available from the corresponding author upon request.
