# Supplementary figures and images for: CD63 negatively regulates hepatocellular carcinoma development through suppression of inflammatory cytokine‐induced STAT3 activation
Source: J Cell Mol Med. 2020 Dec 4;25(2):1024–34. doi: 10.1111/jcmm.16167 (PMC7812266; doi:10.1111/jcmm.16167)

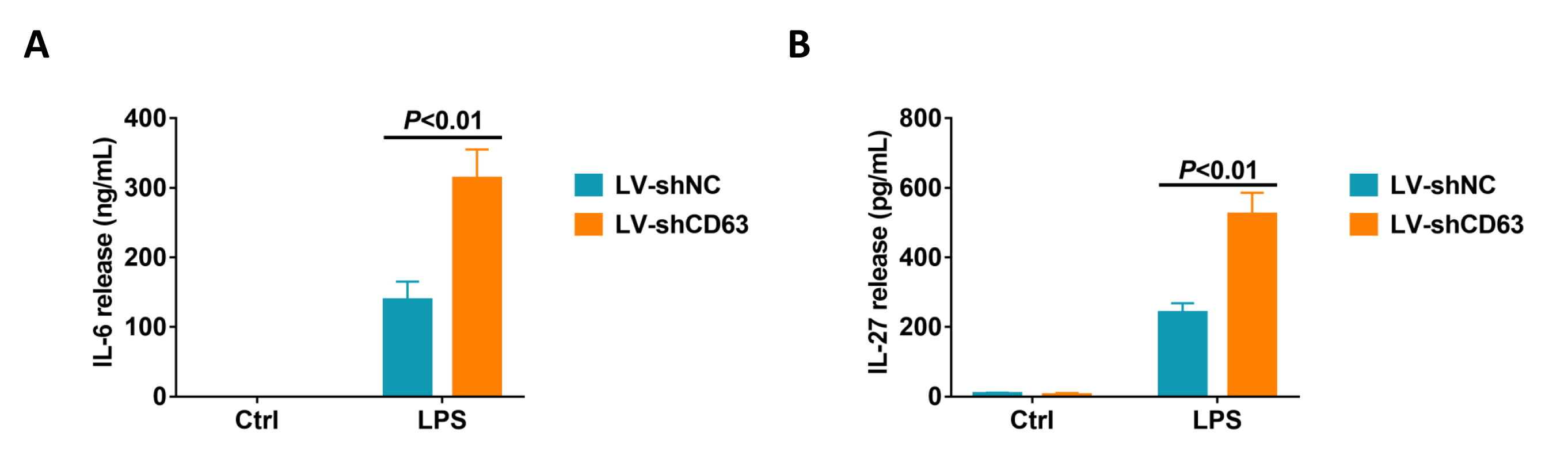

Supplement: Supplementary file 1 — Fig S1 [file JCMM-25-1024-s001.tif]
